# Supplementary material for: Earlier onset of North Atlantic hurricane season with warming oceans
Source: Nat Commun. 2022 Aug 16;13:4646. doi: 10.1038/s41467-022-31821-3 (PMC9381499; doi:10.1038/s41467-022-31821-3)
Supplement: Supplementary file 1 — Supplementary Information [file 41467_2022_31821_MOESM1_ESM.pdf]

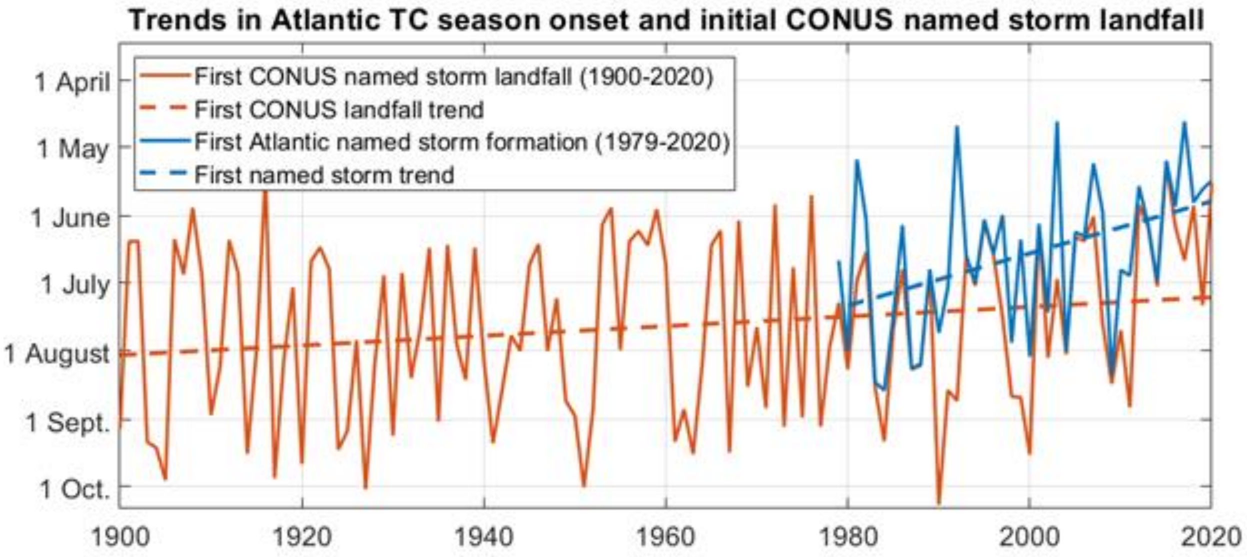


Supplementary Figure 1. **Trends in Atlantic tropical cyclone (TC) season onset.** Dates and linear trends of first Atlantic TC with sustained winds >= 34 knots (1979–2020), and first TC with sustained winds >= 34 knots passing within 0.5° of the continental United States (CONUS) over 1900–2020.

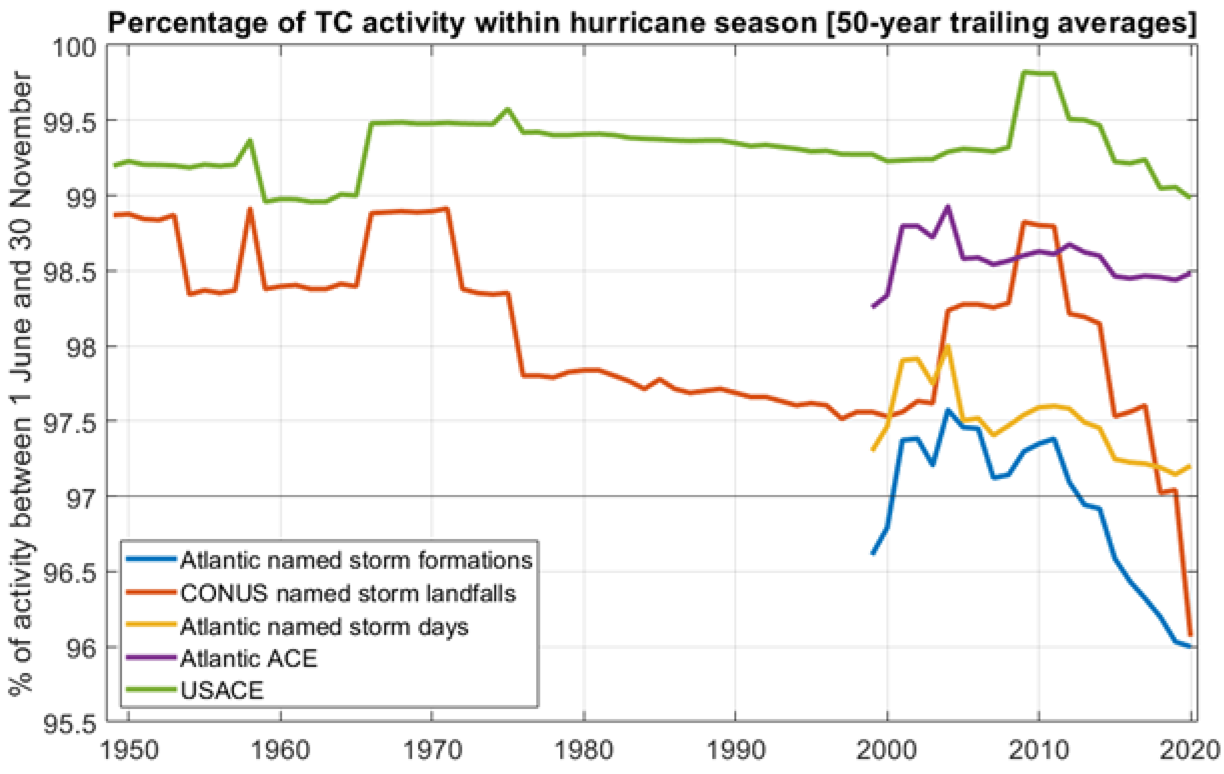


Supplementary Figure 2. **Proportion of tropical cyclone (TC) activity within hurricane season.** Percentages of 50-year trailing averages of named storm formations, continental United States (CONUS) named storm landfalls, named storm days, Atlantic Accumulated Cyclone Energy (ACE), and CONUS ACE (USACE) occurring 1 June–30 November relative to all activity. Timeseries begin in 1900–1949 for landfalls and USACE and 1950–1999 for other parameters.

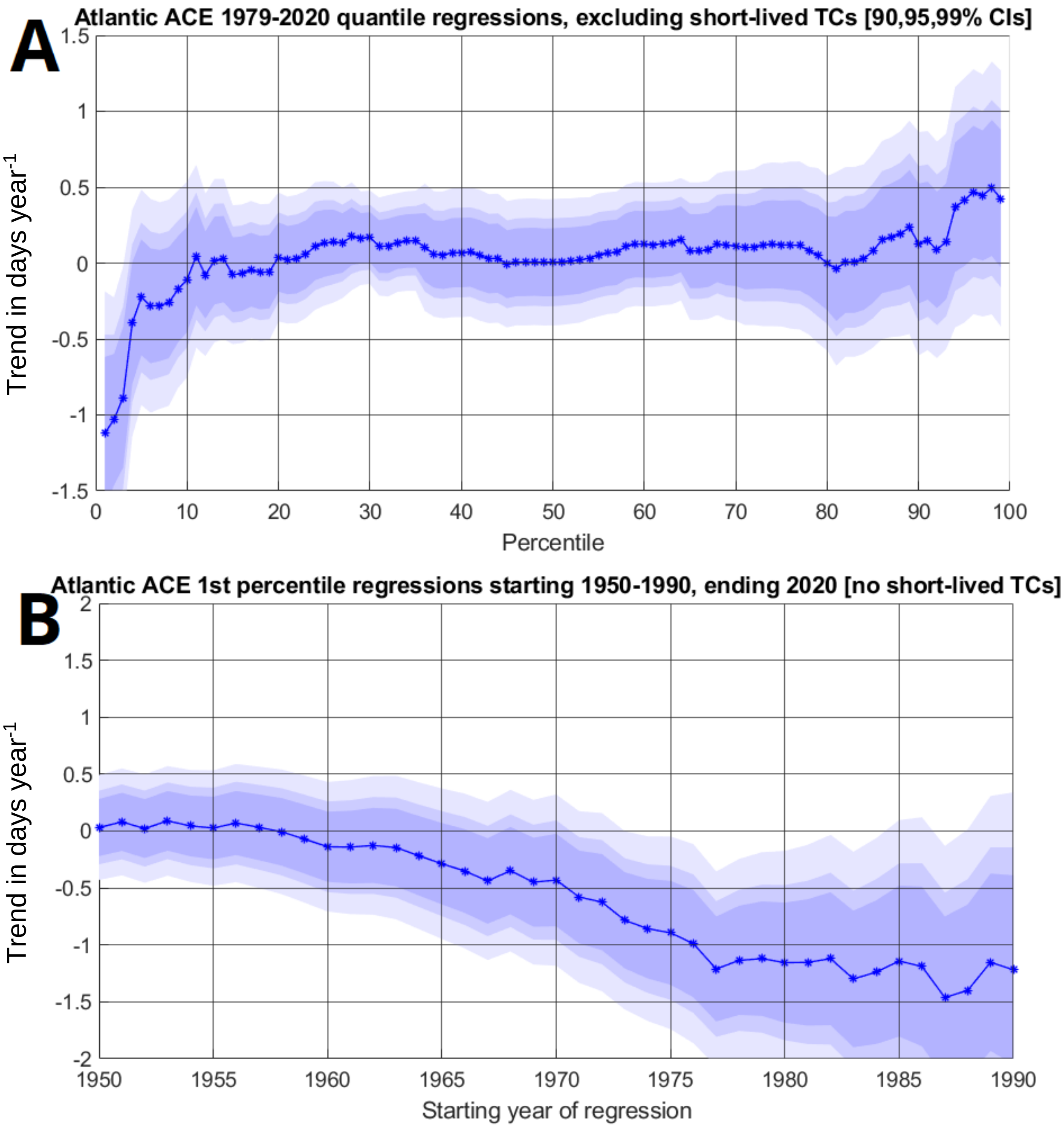


Supplementary Figure 3. **Quantile regressions of Accumulated Cyclone Energy (ACE) and ACE over the continental United States (USACE) with year, excluding short-lived tropical cyclones (TCs)**. As in Fig. 1a and 1b, but substituting Atlantic ACE excluding short-lived TCs.

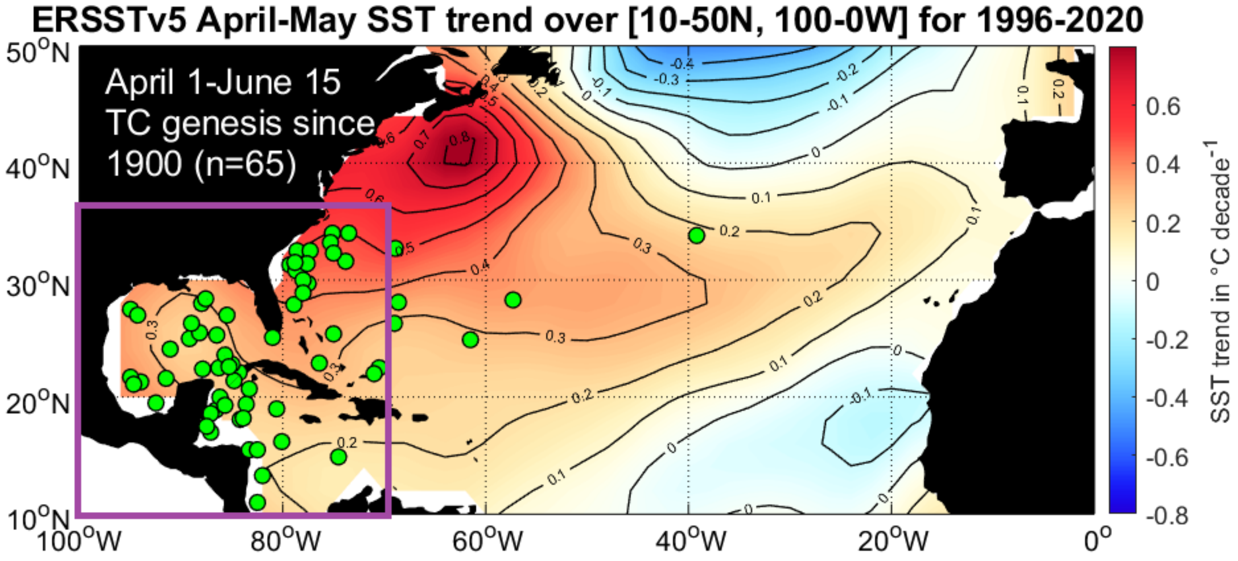


Supplementary Figure 4**. Early-season tropical cyclone (TC) genesis locations and sea surface temperature (SST) trends.** Spatial linear trends in ERSSTv5 April–May mean SST over the North Atlantic basin [10-50°N, 100-0°W] in °C decade$^{-1}$. Points at which an Atlantic TC or subtropical cyclone first reached sustained winds of >=34 knots in HURDAT2 over the period 1 March through 15 June, a timeframe roughly corresponding to the first 1% of Atlantic Accumulated Cyclone Energy (ACE), for years 1900–2020 are shown in green. The outlined box covers 10-36°N, 100-70°W and is the area over which Genesis Potential Index, SST, relative humidity, and shear averages were calculated, excluding the portions over the eastern Pacific for SST calculations. Green dots represent TC formation locations.

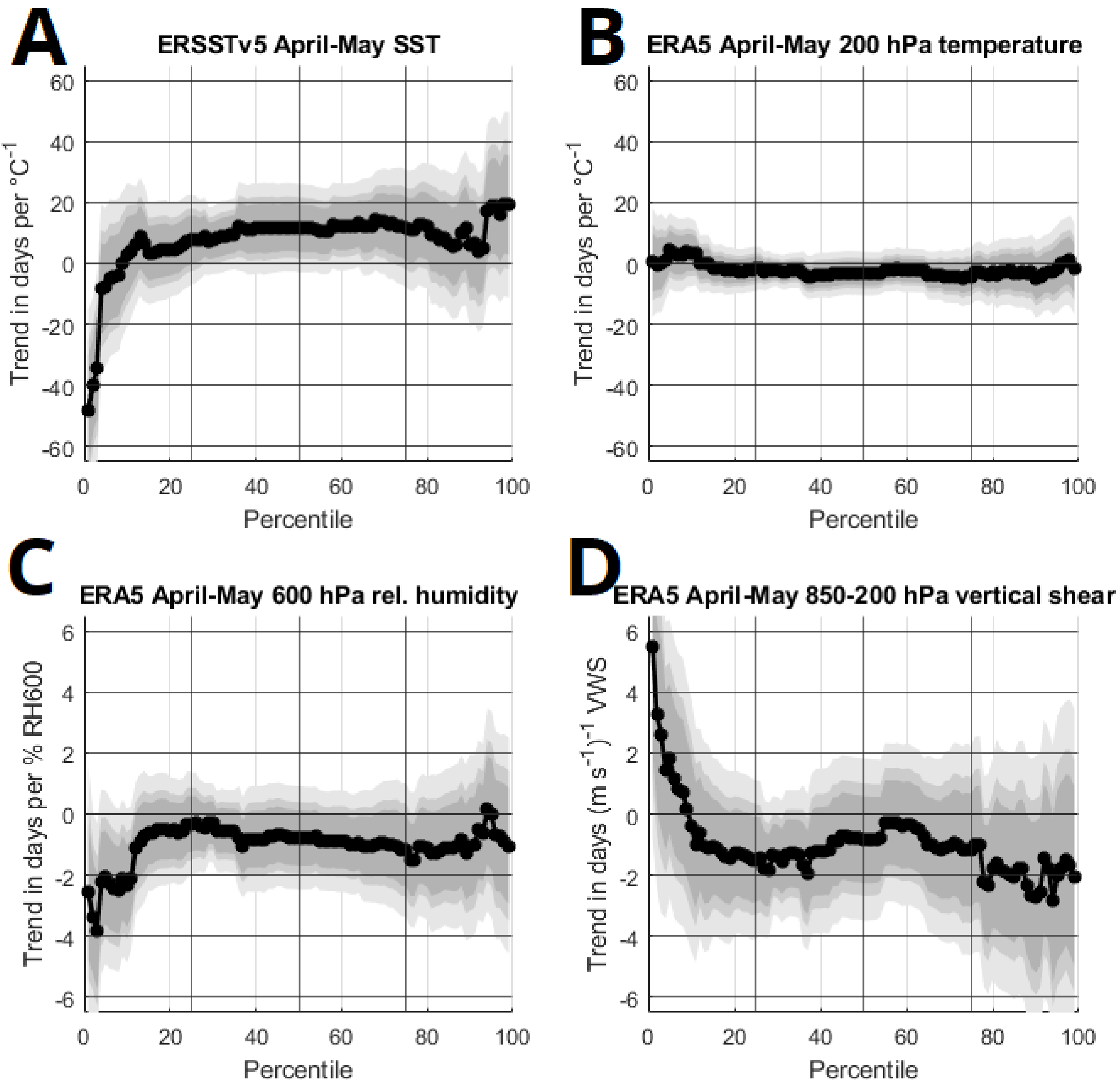


Supplementary Figure 5**. Quantile regressions of Accumulated Cyclone Energy (ACE) with genesis parameters, excluding short-lived tropical cyclones**. **a-d** As in Fig. 3a-d, substituting quantiles of Atlantic ACE and excluding tropical cyclones lasting fewer than 48 hours with a maximum sustained wind at or above 34 knots.

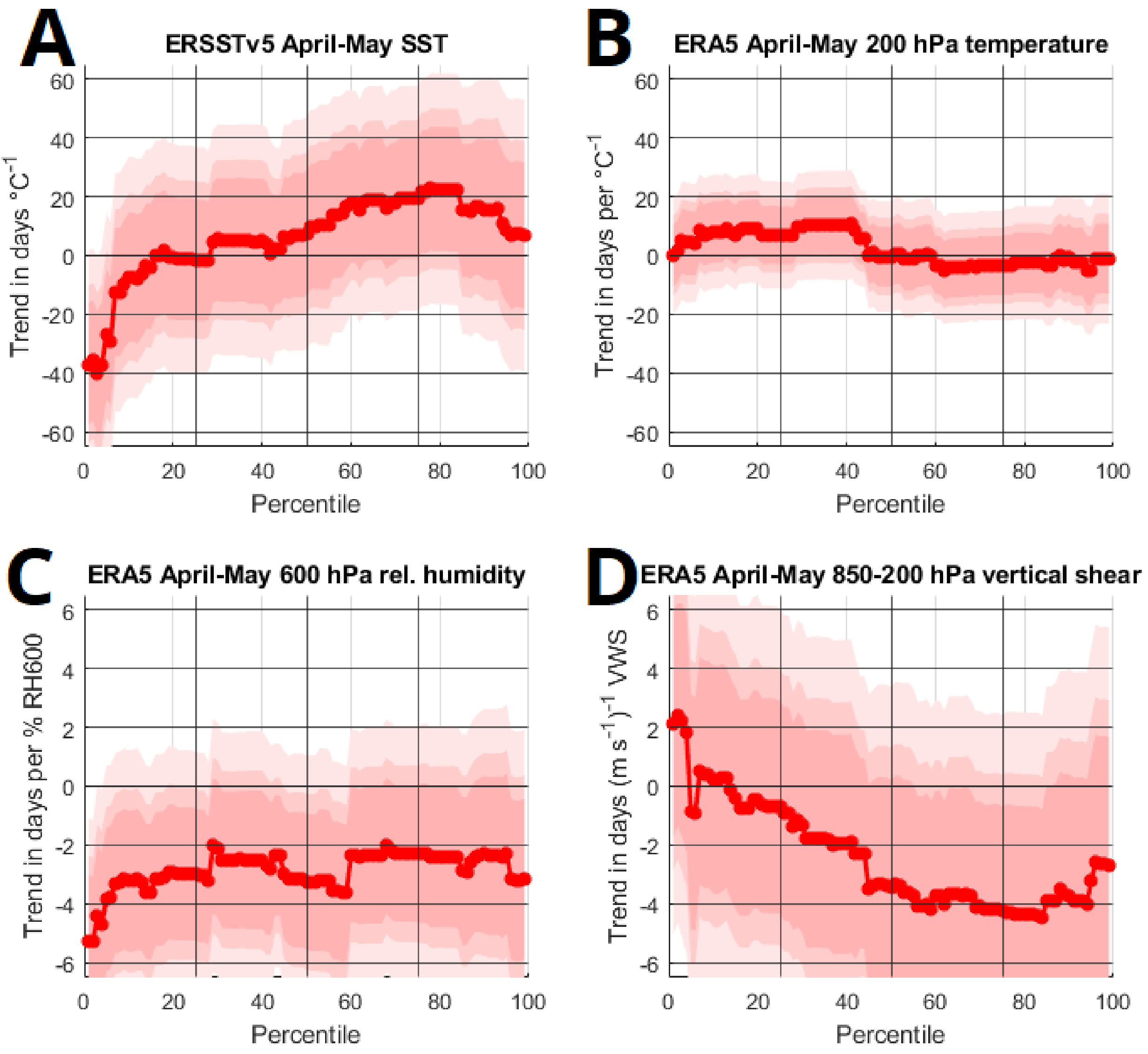


Supplementary Figure 6**. Quantile regressions of continental United States Accumulated Cyclone Energy (USACE) with genesis parameters. a-d** As in Fig. 3a-d, substituting quantiles of continental United States Accumulated Cyclone Energy.

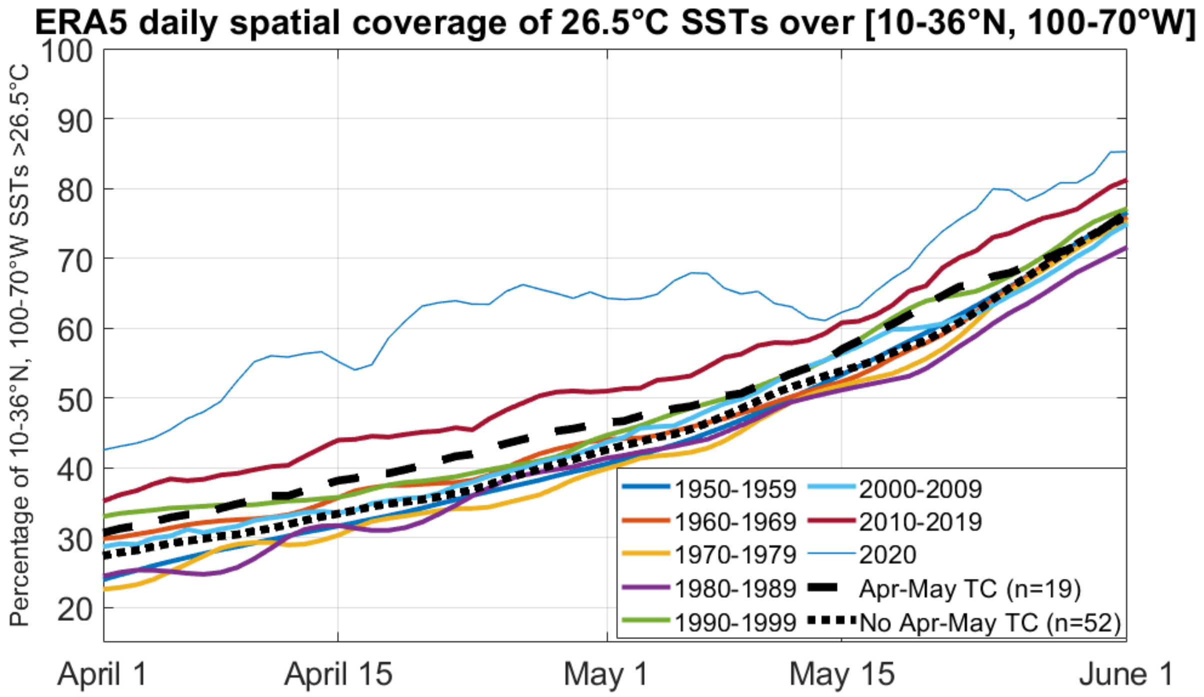


Supplementary Figure 7. **Spring sea surface temperature (SST) threshold coverage.** Percentage of the western Atlantic basin [10-36°N, 100-70°W] with ERA5 daily SSTs exceeding 26.5°C for 1 April–31 May. Coverage shown by decadal means between 1950–1959 and 2010–2019, for 2020, and composites with (dashed) and without (dotted) Atlantic tropical cyclone (TC) genesis during 1 April–31 May over 1950–2020.

| Genesis parameter | 1979-2020 parameter trend (units year$^{-1}$) | 1$^{st}$ %ile ACE regression coefficient (days units$^{-1}$) | 1$^{st}$ %ile USACE regression coefficient (days units$^{-1}$) | 1$^{st}$ %ile ACE threshold date implied shift (days) | 1$^{st}$ %ile USACE threshold date implied shift (days) |
|---|---|---|---|---|---|
| GPI (C07) | **9e-3** | **-30.7** | **-44.2** | **-11.6** | **-16.7** |
| GPI (E10) | 2.7e-7 | **-4.11e5** | **-2.1e-5** | -4.8 | -3.7 |
| SST (°C) | **0.017** | **-34.7** | **-37.0** | **-24.8** | **-26.4** |
| 200T (°C) | 0.016 | -0.8 | -0.2 | -0.5 | -0.13 |
| RH (%) | 5.1e-3 | **-4.8** | **-5.3** | -1.0 | -1.1 |
| VWS (m s$^{-1}$) | -0.052 | 0.96 | 2.1 | -2.1 | -4.5 |

Supplementary Table 1**. Genesis potential index (GPI) and genesis parameter trends and regression coefficients with Accumulated Cyclone Energy (ACE) and continental United States ACE (USACE) first percentile threshold dates.** Average spring GPIs[23,24], sea surface temperatures (SST), 200 hPa temperature (200T), 600 hPa relative humidity (RH), and 850-200-hPa vertical wind shear (VWS) trends over 10–36°N, 100–70°W, quantile regression coefficients for the 1$^{st}$ percentile of ACE and USACE threshold dates, and the implied shift in the 1$^{st}$ percentile ACE and USACE threshold date shifts over 1979–2020. Significant differences from zero at $p<0.05$ are bolded. Shifts given in column four are column one trends multiplied by column two coefficients; shifts given in column five are column one trends multiplied by column three coefficients. GPI[24] data has been smoothed for quality control purposes.
